# 

**Published:** 1890-08-09

**Authors:** 


### NOTICE TO CORRESPONDENTS.

All MS., Letters, Books for Review, and other matters intended for the Editor should be addressed The Editor, The Lodge, Porchester Square, London, W.

The Editor cannot undertake to return rejected M.S., even when accompanied by stamped directed envelope.

**Notice to Advertisers and Subscribers.**

All Advertisements, Orders for Copies of The Hospital, and Business Communications should be addressed to The Publisher, *not to the Editor*, The Hospital, 140, Strand, W.C.

**Bound Volumes of "The Hospital."**

Vol. VI., containing full page portraits of T.R.H. the Prince and Princess of Wales, and Miss Florence Nightingale, is obtainable at 4s., post free.

Orders will be received for Vol. VII., 4s. post free. Cases for binding may be had of the Publisher, at 1s. 6d. each.

**To Residents, Secretaries, and Matrons.**

The Editor will be glad to have the Regulations and each Report as issued by Asylums, Hospitals, Convalescent and Nursing Institutions, as well as those of other Charities, and an early copy in duplicate of all Appeals and Festival Notices, etc., addressed to *The Editor, The Lodge, Porchester Square, London, W.* Any superintendent, secretary, matron, or other chief official who regularly sends such information may be registered, on application to the Publisher, to receive free of cost a cover for binding The Hospital in half-yearly volumes.

### BURDETT'S HOSPITAL ANNUAL FOR 1890

Is now ready, and may be obtained of the Publisher, The Hospital Offices, 140, Strand, W.C., price 2s. 6d. limp boards, or 3s. 6d. cloth. All orders must be accompanied by a remittance.

### General Charities.

[This Column is devoted to an account of work of charitable institutions other than Medical Charities. The Editor will be glad to receive reports or news of work at 140, Strand. *Philanthropy* should be plainly written on the envelope.]

**THE RAGGED SCHOOL UNION** sent away 3,200 children last year. The Union has six "Homes," located severally at Folkestone, Thursley, East Grinstead, Brenchley, Chislehurst, and Caterham, all held under varying tenures, but none of them actually the property of the society; still, the Committee are hoping soon to become possessed of some really their own. The Committee send some children away to cottages, but they think that for special cases the Homes offer advantages not otherwise obtainable, so the majority are sent to them. Mr. John Kirk, the Secretary, tells us that the cost comes out about 5s. a-week per head. The parents are expected to share the expense, and the local school helps too. Friends of the Union last year gave supplies of clothing which helped to make the children tidy before they went away. Besides mere children, van and errand boys and factory girls—in fact, older boys and girls who wanted change, and were no more likely to get it than their younger brothers and sisters—were sent for a holiday, and the voluntary teachers of the school went away, too, with the children when they needed rest. A few days ago in the country, we saw a waggon full of small Londoners in a lane. Their hands were full of ferns, flowers, and roadside treasures generally, they were singing and shouting, and, in spite of the old look on the small faces which told so plainly of a hard life in a stuffy city, there was a look of keen enjoyment about the children which made one wish that rich folk could see the joy that 10s. brings to a London child's heart for a whole fortnight.

**THE VICAR OF ST. MARY'S, PLAISTOW, E.**, sends us a petition for his mission house, day nursery, and nurses' home, and St. Mary's Holiday Home, Southend-on-Sea. The day nursery contains a crêche, and 70 babies and children have been sent off from it, under the care of seven of the nursing sisters of Plaistow, to Southend for a holiday. Twenty thousand people are in this parish of St. Mary's, and Plaistow is a miserable spot and the people very poor. The vicar earnestly asks for more help to be given him. Infant mortality has been terribly high at Plaistow. Who will help to decrease it?

Mr. Barnett, the Vicar of St. Jude's, Whitechapel, is quite right. He says how little the excitement about the terrible murders in that district has accomplished. A small district of about three acres, the Flower and Dean Street area, he describes as "a plague spot." As a great many of the leases have fallen in the freeholders have now no excuse for not doing something to improve this place; but then they don't have to live there, and the souls and bodies of a few people is such a small matter.

The promoters of the concert in aid of the **SOCIETY FOR PREVENTION OF CRUELTY TO CHILDREN** handed in £900 to the institution.

The Musical Drill Classes of the **HACKNEY JUVENILE MISSION** have been a great success. The children skip and jump and do exercises and drills and enjoy it all keenly. These evening drills are twofold in their good—they develop the children's bodies and keep them out of harm's way and the streets as well. This Mission has a Holiday Fund, too, and sends away children at a cost of six shillings for a fortnight per head. How does it manage? It sounds so very cheap. The cheapest cost we know of is five shillings a week for one child.

**THE SOUTH LONDON ASSOCIATION FOR ASSISTING THE BLIND** take their 300 members and their guides for an outing every year to the seaside. All these people are in great poverty, and their delight in a day's change is as great as that of anybody possessed of sight. The Hon. Secretary, Mr. J. T. Edmonds, Carlton Villa, 155, Brixton Road, S.W., will be grateful for any help towards this year's treat.

The sum of £250 is wanted to build a Sunday-school and recreation-room in the Ascension District, Victoria and Albert Docks. There is not room for the children who attend the school, and no place for social gatherings at all.

5,000 volumes are contained in the new library which has been so generously given to Camberwell and Lambeth by Mr. William Minet.

We wish all the odd pennies we see indiscriminate givers showering on begging people in the street could be collected. They would make quite a grand sum for some of our really deserving charities. Six-and-eightpence was found on one woman in February, which was one day's haul! That excellent society, **THE MENDICITY SOCIETY**, tells us that out of 693 persons convicted of begging last year, only two on leaving prison applied to the society for work—the rest infinitely preferred to go back to street begging, which, thanks to foolish givers, is a remunerative and easy profession.

### Scraps and Gleanings.

Hove reports a death-rate of only 9·4 per 1,000.

Leamington Hospital Saturday realised £118.

Cholera is increasing to a terrible extent in Eastern ports.

Derby Infirmary is to be entirely rebuilt at a cost of £50,000.

The village of Bredbury boasts the lowest death-rate in England.

Over £100 was taken in the Cathedral at Oxford on Hospital Sunday.

The poor people of Japan are starving, owing to the failure of the rice crops.

An effort to resuscitate the court of governors of Radcliffe Infirmary has failed.

The Grand Summer Fête in aid of the Gravesend Hospital realised nearly £200.

Dr. Bilhant, of Paris, tells of a case of diphtheria in a man caught from a pigeon.

Sir James Paget has been elected Hon. President of the Berlin Medical Congress.

Canterbury Hospital is going to discontinue the half-yearly meeting, as no governors attend.

Mrs. M. McLean has left £200 each to the Liverpool Royal Infirmary and Home for Nurses.

In New York and Chicago the deaths from sunstroke have lately numbered five or six a day.

Mr. W. J. Crossley offers £5,000 towards the rebuilding of Manchester Infirmary on its present site.

The financial statement of the Greenwich Saturday Night Popular Entertainments is very satisfactory.

Hull Victoria Hospital for Children issues a good report; they hope to open their new buildings this year.

Ardsley has this year gone in for an open-air concert in aid of the Barnsley Becket Hospital. Over £28 was collected.

The working men of Earl's Colne have sent £15 to the Colchester Hospital; most creditable for such a small village.

The French Senate has voted a credit of 100,000 francs for the establishment of sanitary posts to prevent the importation of cholera.

Dr. Sarah H. Stevenson, of Chicago, stayed with Miss Faithful, and inspected the Manchester hospitals, on her way to the Berlin conference.

The *Daily Graphic* of last Friday contained an account and illustrations of the Convalescent Home for Children at Eaglesham, near Glasgow.

The Herbert Convalescent Home at Bournemouth admitted 376 patients last year. The Home has been greatly improved lately, and is economically managed.

The British Medical Association has held very successful meetings at Birmingham, the addresses having been of exceptional interest, and the audiences very large.

Lady Paget discourses in the *National Review* on "Count Mattei's System"—an unprofessional treatment of a subject the profession has wisely refused to recognise.

The Cremation Conference opened at Berlin on August 4th, under Herr Materne. A telegram was sent to Signor Crispi thanking him for his sympathy with the movement.

The government has appointed a commission of five doctors at Cairo to make necessary arrangements in case cholera appears there. The deaths at Mecca from cholera are numerous.

During the quarter ending June last five dogs were seized with rabies in the county of Middlesex, and but for this the Board of Agriculture would probably have withdrawn the muzzling order.

Final arrangements have been made to erect a new infectious diseases hospital, near Gain Lane, Calverley Moor, by the Joint Hospital Board of the Idle, Eccleshill, Calverley, Farsley, and Pudsey districts.

The Bengal Government has appointed a committee to consider the question of a new leper asylum in Calcutta. There were 387 lepers in Calcutta according to the last census, but probably many escaped enumeration, and the number is much larger now.

In a paper on Tuberculosis in Belgium, MM. Destrée and Gallmaerts come to the conclusion as the result of their investigations that, in comparing the mortality from phthisis of bachelors, married men, and widowers, the last are very much more subject to this disease than either of the other classes.

At a meeting of the Johannesburg Sanitary Board Dr. Matthews presented a report showing that during the year ending June 30th, 434 males and 182 females (all white), or 38·5 per thousand of the population, died here. Of this number 124 died from typhoid and 36 from diarrhœa and dysentery.

Lowestoft Hospital is going to exclude typhoid cases, and charge each in-patient a shilling a-week for washing. With all sympathy for the hospital in its poverty, we do not approve of these resolutions; but we heartily commend the ladies of Lowestoft for their vigorous efforts in the disagreeable work of collecting.

The nomination of Leprosy Commissioners has been made in two out of three cases. The council of the Royal College of Surgeons have nominated Mr. Kassack, Fellow of the College. The gentleman appointed by the Leprosy Committee is Dr. Buckmaster, lecturer on physiology at St. George's Hospital Medical School.

At the annual meeting of Belfast Consumption Hospital, Archdeacon Seaver and Mr. Samuel Young reported having visited addresses of fifty-one male patients who had applied for admission between April, 1889, and the 31st May last. Of these they found but seven living, all the rest having died. Dr. W. B. Croker also reports having visited upwards of thirty female patients who had applied for admission during the last four months, and of these he found but five living.

